# Torrefaction and Pyrolysis of Lignocellulosic and Organo-Mineral Pellets: A Comparative Study of Green Coconut Husk and Sewage Sludge

**DOI:** 10.1021/acsomega.6c08411

**Published:** 2026-09-30

**Authors:** Daniel S. Serra, Lucinda O. Amaro, Fladimir L. Gondim, Cássia M. Santos, Cayo C.F. Moraes, Diego M. Rebouças, Mona Lisa M. de Oliveira

**Affiliations:** † Energy Conversion Laboratory, Center of Science and Technology, State University of Ceará, Fortaleza 60714-903, Brazil; ‡ Diamante Energia, Energia Pecém, São Gonçalo do Amarante, Ceará 62670-000, Brazil

## Abstract

Thermochemical conversion is a promising route for the valorization of residual biomass, enabling energy recovery and the production of solid, liquid, and gaseous products. However, direct comparisons between torrefaction and pyrolysis of chemically distinct pelletized residues remain limited, obscuring the influence of feedstock composition on mass and energy partitioning. Here, green coconut husk and sewage sludge pellets were subjected to torrefaction and pyrolysis in the same batch-operated reactor to determine how lignocellulosic and organo-mineral feedstocks respond to increasing thermal severity. Torrefaction was performed at 250 °C, while pyrolysis was conducted at 400 and 600 °C under a nitrogen atmosphere, using 1.0 kg of pellets per process. Green coconut husk pellets showed higher volatile matter and fixed carbon contents than sewage sludge pellets (50.61 ± 4.84% and 30.30 ± 3.92% versus 38.44 ± 3.94% and 7.35 ± 1.14%, respectively), whereas sewage sludge pellets presented higher ash, nitrogen, and sulfur contents (45.85 ± 4.42%, 2.88 ± 0.94%, and 0.70 ± 0.12%, respectively). Increasing thermal severity reduced solid yield and increased volatile product formation. At 600 °C, solid yield decreased to 29.44 ± 1.12% for green coconut husk and 37.12 ± 3.32% for sewage sludge, while non-condensable gas yield reached 28.66 ± 1.85% and 20.27 ± 2.15%, respectively. The solid HHV reached 26.06 ± 1.99 MJ·kg^–1^ for green coconut husk at 400 °C and 18.34 ± 2.26 MJ·kg^–1^ for sewage sludge at 600 °C. At 600 °C, H_2_ content reached 36.12 ± 2.33% and 39.22 ± 3.03% for green coconut husk and sewage sludge, respectively, with H_2_/CO ratios of 2.03 and 2.47, respectively. Torrefaction showed higher solid energy yield at 250 °C, reaching 74.25% for green coconut husk and 74.17% for sewage sludge, indicating better energy retention in the solid fraction. In contrast, pyrolysis promoted broader energy redistribution into condensable liquids and combustible gases. Overall, torrefaction favored solid-phase energy retention, whereas pyrolysis promoted a broader distribution of recovered energy among solid, liquid, and gaseous products.

## Graphical abstract

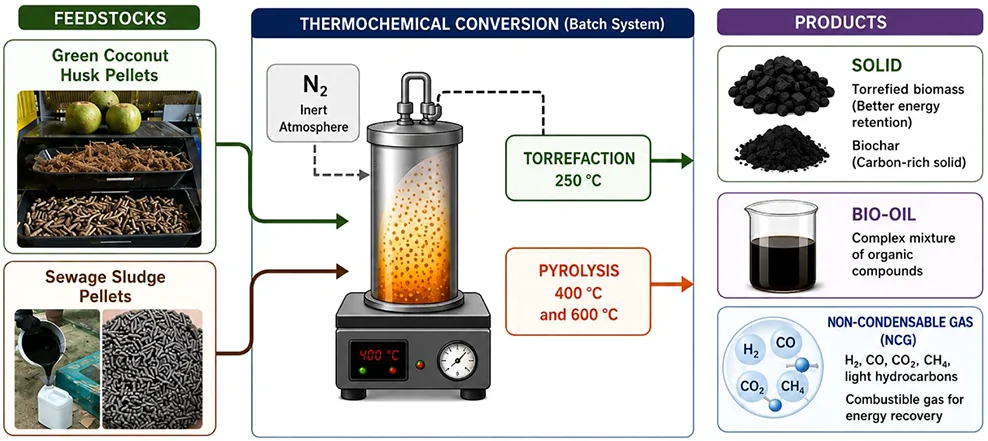

## 1 Introduction

Thermochemical conversion has become an important strategy for the valorization of organic residues, enabling volume reduction, energy recovery, organic matter stabilization, and production of solid, liquid, and gaseous products with different potential applications. In this context, green coconut husk and sewage sludge represent two abundant but chemically distinct residual feedstocks.

Green coconut husk is a lignocellulosic biomass mainly composed of cellulose, hemicellulose, and lignin, which favors its conversion into carbon-rich solids, condensable organic vapors, and combustible gases.^1–5^ Sewage sludge, in contrast, is an organo-mineral residue containing organic matter, ash-forming components, nutrients, and potentially hazardous elements, making its final disposal a technical, sanitary, and environmental challenge.^6–11^

Among thermochemical routes, torrefaction and pyrolysis differ mainly in thermal severity, technological complexity, and target products. Torrefaction is usually conducted between 200 and 300 °C under inert or oxygen-limited conditions and is primarily aimed at upgrading the solid fraction.^12–14^ During this process, drying, partial devolatilization, hemicellulose degradation, and removal of low-molecular-weight oxygenated compounds may occur, resulting in a torrefied solid with lower moisture content, lower volatile matter, reduced hygroscopicity, higher heating value, and improved energy density compared with the raw feedstock.^12,14–16^

Pyrolysis, on the other hand, is conducted at higher temperatures, commonly between 400 and 800 °C, under oxygen-free or oxygen-limited conditions, promoting more extensive thermal decomposition of the feedstock into three main fractions: biochar, bio-oil, and non-condensable gases (NCGs).^17,18^ Biochar may be used as a solid fuel,^5,12^ soil amendment,^1,11,19^ adsorbent,^1^ or carbon sequestration material.^10,11^ Bio-oil contains a complex mixture of organic compounds with potential energetic and chemical applications,^9,20^ while NCG, mainly composed of CO_2_, CO, H_2_, CH_4_, and light hydrocarbons, may be used for thermal energy recovery within the process.^5,17^

However, compared with torrefaction for solid fuel upgrading, pyrolysis requires stricter control of temperature, atmosphere, vapor residence time, condensation, bio-oil recovery, and gas management, making it a more technologically demanding and operationally complex route, especially when liquid and gaseous products are targeted.^9,17,21,22^

Previous studies have investigated sewage sludge pyrolysis under different temperature and residence time conditions,^6,10^ sewage sludge torrefaction over the 200–300 °C range,^12^ and the pyrolysis of green coconut husk pellets at 400 and 600 °C.^5^ More recently, the co-pyrolysis of sewage sludge and coconut shell has been investigated, providing insight into the interactions and mechanisms occurring when both feedstocks are thermochemically converted as a mixture.^23^ Despite these advances, direct comparisons between chemically distinct pelletized residues subjected to torrefaction and pyrolysis under equivalent experimental conditions, including their mass and energy partitioning, remain limited. Because existing studies involve different feedstocks and operating conditions, comparisons across independent experiments make it difficult to clearly separate the influence of feedstock composition from that of process conditions. A controlled comparison using the same reactor and experimental protocol can therefore provide a clearer assessment of how lignocellulosic and organo-mineral feedstocks respond to increasing thermal severity.

Green coconut husk and sewage sludge were selected to represent two chemically contrasting residual matrices, a lignocellulosic agricultural residue and an organo-mineral residue, while maintaining a comparable pelletized physical form. Accordingly, the central question addressed in this study was whether these contrasting feedstock compositions would lead to different mass and energy partitioning responses when torrefaction and pyrolysis were performed under equivalent experimental conditions. The study therefore evaluated how increasing thermal severity affected product yields, the physicochemical and energetic properties of the solid products, the energetic properties of the condensable liquid and non-condensable gas fractions, gas composition, and overall energy distribution.

## 2 Materials and Methods

### 2.1 Biomass Feedstocks

Green coconut husks were collected at Fazenda Grangeiro, Paracuru, Ceará, Brazil. The biomass was cut and shredded using a TRF 70 forage shredder (Trapp, Brazil). It was then dried for 4 days in a typical small-scale movable drying structure under natural solar radiation and convective airflow. After drying, the material was milled to obtain a mixture composed of powder and fibers, which was subsequently processed into pellets using a pelletizer (Pellet Machine-SKJ 200, Zeromskiego, Poland). The moisture content was adjusted to approximately 15–20% to ensure proper compaction during pelletization.

The sewage sludge used in this study was obtained from an upflow anaerobic sludge blanket (UASB) reactor at the wastewater treatment plant, operated by the public water and sanitation utility of the state of Ceará, Brazil (CAGECE). At the treatment plant, the sludge was conditioned in drying beds and, after drying, was directly pelletized using the same pelletization system adopted for the green coconut husk.

After pelletization, pellets from both feedstocks had similar dimensions, with pellets of approximately 10 mm in diameter and 20–30 mm in length. Thus, two chemically distinct residual matrices, a lignocellulosic agricultural residue represented by green coconut husk and an organo-mineral urban residue represented by sewage sludge, were processed in a comparable physical form before torrefaction and pyrolysis.

### 2.2 Biomass Torrefaction and Pyrolysis System

Torrefaction and pyrolysis were performed in a batch-operated biomass torrefaction and pyrolysis system (BTPS), as the same experimental unit was used for both thermal processes by adjusting the final operating temperature (Figure 1).

**Figure 1:**
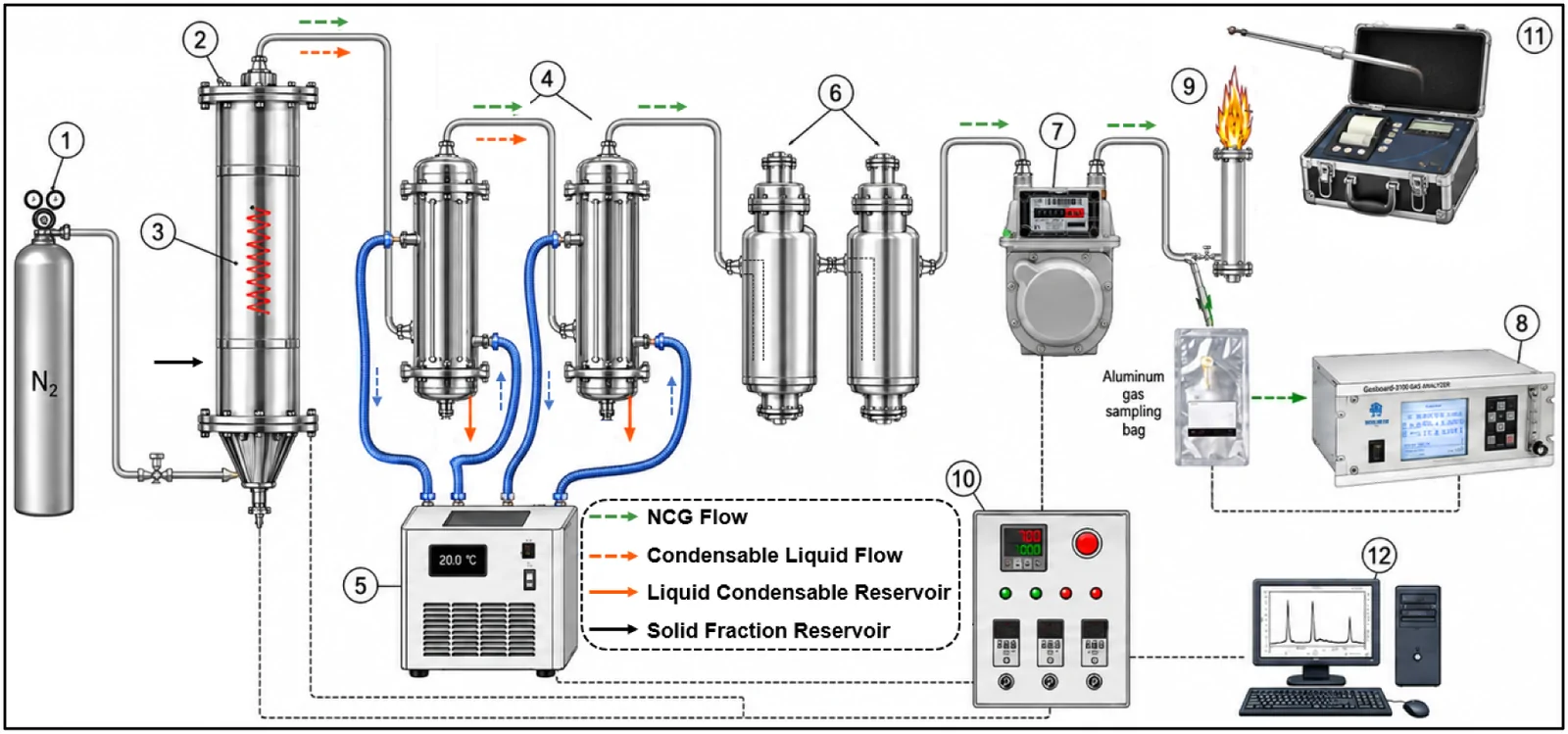
Schematic representation of the biomass torrefaction and pyrolysis system (BTPS) used for product recovery and gas analysis: (1) N_2_ cylinder; (2) fixed-bed torrefaction/pyrolysis reactor; (3) electric heating resistance; (4) condensers in series; (5) thermostatic bath; (6) gas-washing vessels in series; (7) gas meter; (8) gas analyzer; (9) flare; (10) control panel; (11) combustion gas analyzer; and (12) data acquisition.

For each experiment, 1000 g of pellets was loaded into the reactor, and each experimental condition was evaluated in three independent runs for both green coconut husk and sewage sludge pellets. Three final temperatures were evaluated: 250 °C for torrefaction (T250) and 400 and 600 °C for pyrolysis (P400 and P600, respectively). These temperatures were selected as representative thermal conversion conditions based on previous studies.^5,6,10^ A heating rate of 20 °C·min^–1^ was selected as a moderate heating rate within the range employed for slow pyrolysis, while a residence time of 60 min at the final temperature was applied uniformly to all experimental conditions. The same heating rate, residence time, and N_2_ flow rate were maintained across both feedstocks and all thermal conditions to provide a standardized basis for evaluating the effects of feedstock composition and thermal severity. Figure 1 illustrates the operational flow diagram of the biomass torrefaction and pyrolysis system used in the thermal conversion processes.

The process started with the supply of nitrogen from the N_2_ cylinder (Figure 1–1) at a flow rate of 500 mL·min^–1^ to purge the system and maintain an inert atmosphere inside the fixed-bed reactor (Figure 1–2). The reactor was constructed of stainless steel, operated in batch mode at atmospheric pressure and had internal dimensions of 500 mm in height and 100 mm in diameter. Heating was provided by a 4000 W electric heater (Figure 1–3), positioned around the reactor and covered by thermal insulation to reduce heat losses and improve thermal stability during the process. The temperature profile and heating rate were controlled using a PID controller (Novus N1020), integrated into a control panel (Figure 1–10).

During thermal conversion, the solid feedstock remained inside the reactor, while the generated vapors and gases were carried by the nitrogen flow to the downstream recovery system. The condensable fraction released during torrefaction and pyrolysis was directed to a multistage condensation system. The primary condensation stage consisted of two identical cylindrical condensers arranged in series (Figure 1–4). Both condensers were cooled by water supplied from a thermostatic bath (solid steel) (Figure 1–5), which maintained the cooling fluid at 10 °C. This system allowed the recovery of the condensable liquid fraction produced during the thermal conversion process.

After passing through the condensers, the remaining gas stream, comprising non-condensable gases (NCG), was directed to two gas-washing bottles arranged in series (Figure 1–6). These washing bottles contained water and were used to retain remaining volatile compounds that were not fully condensed in the previous stage. The NCG stream was then quantified using a gas meter (Figure 1–7). In each independent experimental run, a 1 L NCG sample was collected in an aluminum gas sampling bag for subsequent compositional analysis. Thus, three independent gas samples were obtained for each feedstock and thermal condition, with sampling performed at the final process temperature (250, 400, or 600 °C). The collected samples were analyzed using a multigas analyzer with TCD/ECD/NDIR (Gasboard-3100, Cubic Instruments®) (Figure 1–8). The analyzer quantified H_2_ using a thermal conductivity detector (TCD), while CO, CO_2_, C_2_H_4_ and CH_4_ were measured using non-dispersive infrared sensors. After quantification and compositional analysis, the remaining NCG stream was directed to a flare (Figure 1–9) for combustion.

The combustion of the NCG was evaluated directly at the flare outlet of the experimental system. The exhaust gases generated during NCG combustion were analyzed using a Chemist 900 gas analyzer (Seitron®, Italy) (Figure 1–11). The analyzer monitored O_2_, CO, NO, NO_2_, SO_2_, CO_2_, CH_4_, NO_*x*_, and exhaust gas temperature.

The solid fraction obtained at 250 °C was referred to as torrefied solid, while the solid fractions obtained at 400 and 600 °C were referred to as biochar. The condensable liquid fraction and the NCG were quantified for both torrefaction and pyrolysis, allowing comparison of product distribution across the evaluated thermal severity range.

### 2.3 Feedstock, Torrefied Solid, and Biochar Characterization

Proximate chemical analyses were performed on the raw feedstocks, feedstock pellets, torrefied solids obtained at 250 °C, and biochars produced by pyrolysis at 400 and 600 °C. Proximate analysis was performed in triplicate to determine moisture content (%*M*), volatile matter (%*VM*), ash content (%*A*), and fixed carbon (%*FC*). Moisture content was determined according to ISO 18134–1:2022.^24^ Volatile matter and ash content were determined by instrumental proximate analysis using a thermogravimetric analyzer, following ASTM D7582-24.^25^ Fixed carbon content was calculated by difference after determining moisture, volatile matter, and ash content. The same analytical procedures were applied to the raw feedstocks, feedstock pellets, torrefied solids, and biochars.

The pellet density was determined before thermal conversion. The pellet mass was measured using an analytical balance, and volume was estimated from pellet dimensions, with length and diameter measured using a digital caliper. At least ten pellets from each feedstock were measured to ensure representativeness.

Elemental analyses were performed on the raw feedstocks, feedstock pellets, torrefied solids, and biochars. Carbon, hydrogen, and nitrogen contents were determined according to ASTM D5373,^26^ whereas the sulfur content was determined according to ASTM D4239.^27^ Oxygen content was calculated by difference. The higher heating value (HHV) of the raw feedstocks, feedstock pellets, torrefied solids, and biochars was determined using a bomb calorimeter (Model 1341; Parr Instrument Company), allowing the evaluation of the energy released during complete combustion.

### 2.4 Mass and Energy Balance

The mass and energy balances were determined for both torrefaction and pyrolysis processes using procedures previously applied to pelletized biomass thermochemical conversion systems,^5,28^ allowing comparison between the two feedstocks and the three thermal conditions. For each experiment, the inlet mass corresponded to the mass of pellets loaded into the reactor, while the outlet streams were defined as the solid product, condensable liquid fraction, non-condensable gases (NCG), and process losses. The solid product obtained at 250 °C was referred to as torrefied solid, whereas the solid products obtained at 400 and 600 °C were referred to as biochars. The condensable fraction was recovered after the condensation system, while the remaining gaseous stream was quantified as NCG.

The mass yield of each product was calculated according to eq 1:

1 $$Y_{i}\left(\%\right)=\frac{M_{i}}{M_{0}}\cdot 100$$

where *Y*_*i*_ is the mass yield of product i, *M*_*i*_ is the mass of the solid product, condensable liquid fraction, or NCG, and *M*_*0*_ is the initial mass of pellets loaded into the reactor.

The overall mass balance was calculated according to eq 2:

2 $$M_{0}=M_{s}+M_{l}+M_{g}+M_{loss}$$

where *M_s_* is the mass of the solid product, *M*_*l*_ is the mass of the condensable liquid fraction,*M_g_* is the mass of non-condensable gases, and *M_loss_* represents the unaccounted mass losses associated with incomplete condensation, retention of vapors in the lines, gas leakage, or experimental handling losses.

The percentage of mass loss was calculated according to eq 3:

3 $$Y_{loss}\left(\%\right)=100-\left(Y_{s}+Y_{l}+Y_{g}\right)$$

where *Y_s_*, *Y_l_*, and *Y_g_* are the percentage yields of the solid, liquid, and gaseous fractions, respectively.

Mass balance closure was calculated as the percentage of the initial feedstock mass recovered as solid, condensable liquid, and NCG products.

The mass of the condensable liquid fraction was determined gravimetrically after collection. For the gaseous fraction, the total gas volume was measured during each experiment using the gas meter. The cumulative volume of nitrogen supplied to the system was subtracted from the measured total gas volume to obtain the net NCG volume generated by the feedstock. The volume of each gas species was then calculated from its volumetric concentration, according to eq 4:

4 $$V_{i}=y_{i}\cdot V_{g}$$

where *V_i_* is the volume of each gas species, *y_i_* is the volumetric fraction of the gas species in the NCG stream, and *V_g_* is the net volume of NCG generated during the process after subtraction of the cumulative nitrogen volume supplied to the system.

The mass of each gas species was calculated using the ideal gas relationship, according to eq 5:

5 $$m_{i}=\frac{V_{i}}{V_{m}}\cdot Mmol_{i}$$

where *m_i_* is the mass of gas species i, *V_i_* is its volume, *V_m_* is the molar volume of an ideal gas under the adopted reference conditions, and *Mmol_i_* is the molar mass of the gas species.

The total NCG mass was then obtained by summing the masses of the quantified gas species, according to eq 6:

6 $$M_{g}=\sum m_{i}$$

The estimated species-specific yield of each gas was calculated by dividing its estimated mass by the initial feedstock mass and was expressed as g·kg^–1^ of feedstock. The energy balance was performed by comparing the energy entering the system with the energy associated with the recovered products and the thermal losses. The energy input from the feedstock was calculated using eq 7:

7 $$E_{0}=HHV_{0}\cdot M_{0}$$

where *E*_0_ is the energy content of the initial feedstock, *HHV*_0_ is the higher heating value of the pellets, and *M*_0_ is the initial mass of pellets.

The external electrical energy input was determined from the total electricity consumed by the system during each process, including the reactor heating system, condensation/cooling system, control panel, data acquisition system, and auxiliary components when applicable. This value was recorded in kilowatt hours and converted to the same energy unit adopted for the balance during data processing.

The total energy input of the system was calculated according to eq 8:

8 $$E_{in}=E_{0}+E_{el}$$

where *E_in_* is the total energy input, *E*_0_ is the chemical energy of the feedstock, and *E_el_* is the external electrical energy consumed during the process.

The energy contents of the solid and condensable liquid fractions were calculated according to eqs 9 and 10:

9 $$E_{s}=HHV_{s}\cdot M_{s}$$

10 $$E_{l}=HHV_{l}\cdot M_{l}$$

where *E_s_* and *E_l_* are the energy contents of the solid and liquid fractions, respectively; *HHV_s_* and *HHV_t_* are their respective higher heating values; and *M_s_* and *M_l_* are their respective masses.

The energy content of the NCG was calculated from its lower heating value and total volume, according to eq 11:

11 $$E_{g}=LHV_{g}\cdot V_{g}$$

where *E_g_* is the energy content of the non-condensable gases, *LHV_g_* is the lower heating value of the NCG mixture, and *V_g_* is the total gas volume generated during the experiment.

The *LHV_g_* of the gas mixture was calculated from the volumetric fraction and the lower heating value of each combustible gas species, according to eq 12:

12 $$LHV_{g}=\sum \left(y_{i}\cdot LHV_{i}\right)$$

where *y_i_* is the volumetric fraction of each combustible gas and *LHV_i_* is the lower heating value of the corresponding pure gas.

The energy yield of each recovered product was calculated according to eq 13:

13 $$EY_{i}\left(\%\right)=\frac{E_{i}}{E_{0}}\cdot 100$$

where *EY_i_* is the energy yield of the solid, liquid, or gaseous fraction, *E_i_* is the energy content of each product, and *E*_0_ is the energy content of the initial feedstock.

The global energy balance of the process was expressed according to eq 14:

14 $$E_{in}=E_{s}+E_{l}+E_{g}+E_{loss}$$

where *E_in_* is the total energy input previously defined in eq 8; *E_s_*, *E_l_*, and *E_g_* are the energy contents of the recovered solid, liquid, and gaseous products, respectively; and *E_loss_* corresponds to the non-recovered energy, including heat losses through the reactor wall, condensation system, gas lines, and other system components.

Reactor heat losses were estimated using a heat loss calculation approach described in the literature and adapted to the operating conditions of the present batch system.^29^ After accounting for the energy recovered in the products and the estimated reactor heat losses, the remaining non-recovered energy was reported as residual system heat losses, encompassing heat rejected by the condensation system and other unquantified thermal losses through gas lines, connections, and auxiliary components. This residual term was used to close the global energy balance.

Energy balance closure was calculated as the ratio of the total accounted energy output to the total energy input. Because the residual system heat losses were determined by difference, the global energy balance was close to 100% for all experimental conditions.

In addition to the mass and energy balances, two solid-phase energy indicators were calculated. The energy densification ratio (EDR) was calculated as the ratio between the HHV of the solid product and the HHV of the corresponding pellets. The solid energy yield (SEY) was calculated as the product of the solid mass yield and the EDR, representing the percentage of the initial pellet energy retained in the solid fraction.

### 2.5 Statistical Analysis

Each experimental condition was evaluated in three independent runs (n = 3). The analytical results reported for each condition correspond to samples obtained from these independent experimental runs. Results are expressed as mean ± standard deviation. Statistical differences among groups were evaluated by one-way analysis of variance (ANOVA), followed by Tukey’s honestly significant difference (HSD) post hoc test. Differences were considered statistically significant at p < 0.05. Statistical analyses were performed using GraphPad Prism for Windows (GraphPad Software, Boston, MA, USA).

## 3 Results and Discussion

The results are presented and discussed considering the combined effects of feedstock type and thermal conversion conditions. Green coconut husk pellets and sewage sludge pellets were first compared in terms of their physicochemical and energetic properties, since differences in lignocellulosic composition, ash content, and organo-mineral structure directly influence their thermal behavior. Subsequently, the effects of torrefaction at 250 °C and pyrolysis at 400 and 600 °C were evaluated based on product distribution, solid product characterization, condensable liquid fraction, non-condensable gas composition, and energy balance.

### 3.1 Feedstock, Torrefied Solid, and Biochar Characterization

Table 1 presents the proximate analysis, elemental composition, and pellet density of raw and pelletized green coconut husk and sewage sludge before thermochemical conversion. The two feedstocks had distinct compositions.

**Table 1:** Proximate Analysis, Elemental Composition, and Pellet Density of Raw and Pelletized Green Coconut Husk and Sewage Sludge before Thermochemical Conversion^a^

Parameter	Raw Green Coconut Husk	Green Coconut Husk Pellets	Raw Sewage Sludge	Sewage Sludge Pellets
Proximate Analysis (%)				
Moisture	14.88 ± 2.24^b^	8.87 ± 1.88^a^	11.60 ± 2.74^ab^	8.36 ± 1.78^a^
Volatile matter	56.20 ± 5.78^b^	50.61 ± 4.84^ab^	40.31 ± 4.24^a^	38.44 ±3 .94^a^
Ash	6.93 ± 1.88^a^	10.22 ± 2.30^a^	42.14 ± 4.62^b^	45.85 ± 4.42^b^
Fixed carbon	22.00 ± 2.38^b^	30.30 ± 3.92^c^	5.95 ± 0.84^a^	7.35 ± 1.14^a^
Elemental Analysis (%)				
Carbon	42.36 ± 4.62^c^	32.49 ± 3.80^b^	15.97 ± 1.74^a^	11.54 ± 1.42^a^
Hydrogen	5.46 ± 0.82^a^	4.47 ± 0.92^a^	3.99 ± 0.80^a^	3.86 ± 0.72^a^
Nitrogen	0.22 ± 0.08^a^	0.16 ± 0.04^a^	3.33 ± 0.64^b^	2.88 ± 0.94^b^
Sulfur	0.04 ± 0.01^a^	0.04 ± 0.01^a^	0.75 ± 0.14^b^	0.70 ± 0.12^b^
Oxygen	35.08 ± 2.98^b^	37.72 ± 2.84^b^	22.22 ± 1.94^a^	23.44 ± 2.04^a^
Physical Properties				
Pellet density (kg/m^3^)	----	1386 ± 94^a^	----	1228 ± 82^b^

a Values are expressed as mean ± standard deviation. Comparisons were performed across all four material groups within each row. Means in the same row followed by the same lowercase letter do not differ significantly from each other, whereas different lowercase letters indicate significant differences according to Tukey’s test (p < 0.05).

Green coconut husk had higher volatile matter and fixed carbon contents than sewage sludge, in both raw and pelletized forms, confirming its lignocellulosic character and a greater organic fraction available for thermal decomposition. In contrast, sewage sludge showed much higher ash content, reaching 45.85 ± 4.42% in the pellets, compared with 10.22 ± 2.30% for green coconut husk pellets. This high mineral fraction is typical of sewage sludge and directly affects its behavior during torrefaction and pyrolysis, favoring higher solid residue retention but limiting its energetic quality as a solid fuel, particularly because of the high ash content.^6,12,17,30^

The elemental analysis reinforced these differences between the feedstocks. Green coconut husk showed a higher carbon content than sewage sludge, especially in the raw material (42.36 ± 4.62% and 15.97 ± 1.74%, respectively), while sewage sludge had much higher nitrogen and sulfur contents in the pelletized form (2.88 ± 0.94% N and 0.70 ± 0.12% S) than green coconut husk pellets (0.16 ± 0.04% N and 0.04 ± 0.01% S). These results are consistent with the organo-mineral nature of sewage sludge, which has been described as a complex mixture of organic matter, microorganisms, inorganic material, nutrients, and mineral elements.^8,17^ In contrast, the higher carbon content of green coconut husk favors its conversion into carbonaceous solid products and energy-rich volatile fractions during thermochemical processing, which is consistent with its lignocellulosic composition and with the role of cellulose, hemicellulose, and lignin in the formation of bio-oil, non-condensable gases, and biochar, respectively.^5^

Table 2 summarizes the proximate and elemental compositions of the torrefied solids and biochars obtained from green coconut husk and sewage sludge pellets under different thermal conversion conditions.

**Table 2:** Proximate Analysis and Elemental Composition of Torrefied Solids and Biochars Produced from Green Coconut Husk and Sewage Sludge Pellets under Different Thermal Conversion Conditions^a^

Feedstock	Green Coconut Husk			Sewage Sludge		
Condition	T250	P400	P600	T250	P400	P600
Proximate Analysis (%)						
Moisture	8.45 ± 1.79^b^	5.90 ± 1.25^ab^	4.01 ± 0.85^a^	8.28 ± 1.76^b^	6.16 ± 1.31^ab^	4.18 ± 0.90^a^
Volatile matter	39.01 ± 3.73^d^	21.84 ± 2.09^b^	17.84 ± 1.71^ab^	30.81 ± 3.16^c^	18.37 ± 1.88^ab^	15.00 ± 1.55^a^
Ash	10.80 ± 2.43^a^	11.93 ± 2.64^a^	12.73 ± 2.86^a^	50.38 ± 4.86^b^	59.27 ± 5.71^bc^	63.24 ± 6.12^c^
Fixed carbon	41.74 ± 5.40^b^	60.33 ± 7.80^c^	65.42 ± 8.46^c^	10.53 ± 1.63^a^	16.21 ± 2.51^a^	17.57 ± 2.74^a^
Elemental Analysis (%)						
Carbon	44.38 ± 5.19^b^	72.78 ± 8.51^c^	76.13 ± 8.90^c^	21.81 ± 2.38^a^	35.77 ± 3.90^ab^	37.42 ± 4.08^ab^
Hydrogen	2.96 ± 0.61^a^	3.00 ± 0.62^a^	2.40 ± 0.49^a^	2.56 ± 0.48^a^	2.59 ± 0.48^a^	2.07 ± 0.39^a^
Nitrogen	0.30 ± 0.07^a^	0.77 ± 0.19^a^	0.78 ± 0.20^a^	5.40 ± 1.76^a^	13.86 ± 4.52^b^	14.04 ± 4.58^b^
Sulfur	0.04 ± 0.01^a^	0.05 ± 0.01^a^	0.08 ± 0.02^a^	0.70 ± 0.12^b^	0.88 ± 0.15^b^	1.40 ± 0.24^c^
Oxygen	24.00 ± 1.81^d^	5.58 ± 0.42^b^	0.86 ± 0.06^a^	14.91 ± 1.30^c^	3.47 ± 0.30^b^	0.53 ± 0.05^a^

a T250 corresponds to the torrefied solid obtained at 250 °C, while P400 and P600 correspond to the biochars produced at final pyrolysis temperatures of 400 and 600 °C, respectively. Values are expressed as mean ± standard deviation (n=3). Comparisons were performed across all feedstock–thermal condition groups within each row. Means in the same row followed by the same lowercase letter do not differ significantly from each other, whereas different lowercase letters indicate significant differences according to Tukey’s test (p < 0.05).

Increasing the thermal severity from torrefaction to pyrolysis promoted a clear reduction in moisture and volatile matter for both feedstocks. In green coconut husk, moisture decreased from 8.45 ± 1.79% at T250 to 4.01 ± 0.85% at P600, while volatile matter decreased from 39.01 ± 3.73% to 17.84 ± 1.71%. A similar trend was observed for sewage sludge, with volatile matter decreasing from 30.81 ± 3.16% at T250 to 15.00 ± 1.55% at P600. This behavior is consistent with progressive volatile release and thermal decomposition as temperature increases, as also reported in previous studies on pelletized lignocellulosic biomass and sewage sludge pyrolysis.^5,17,28^

The solid products from green coconut husk showed stronger carbon enrichment than those from sewage sludge. In green coconut husk, fixed carbon increased from 41.74 ± 5.40% at T250 to 65.42 ± 8.46% at P600, and elemental carbon increased from 44.38 ± 5.19% to 76.13 ± 8.90%. In contrast, sewage sludge biochars showed lower fixed carbon values, reaching only 17.57 ± 2.74% at P600, mainly because of the high ash content, which increased from 50.38 ± 4.86% at T250 to 63.24 ± 6.12% at P600. This confirms that the lignocellulosic matrix favored the formation of a carbon-rich biochar, whereas the organo-mineral nature of sewage sludge led to the concentration of inorganic matter in the solid fraction, as also reported for sewage sludge thermal conversion.^6,12^

The high mineral fraction of sewage sludge may also influence pyrolysis pathways beyond the concentration of inorganic matter in the solid product. A comparison between raw and demineralized sewage sludge showed that inherent minerals affected both volatile release and char development and reduced the apparent activation energy in the 300–500 °C range.^31^

The presence of these minerals favored the release of lighter hydrocarbons relative to heavier hydrocarbons and aromatic compounds, indicating a catalytic contribution to secondary cracking reactions. More recent evidence indicates a dual role of sewage sludge ash, in which the inorganic fraction can hinder heat transfer while simultaneously catalyzing reactions such as C–C bond cleavage, decarboxylation, and dehydration.^32^

The elemental analysis also showed important differences in heteroatom retention. Sewage sludge solids showed numerically higher nitrogen and sulfur contents than green coconut husk solids in all conditions, with significant increases in nitrogen content mainly observed at P400 and P600, reaching 14.04 ± 4.58% N at P600. The marked increase in nitrogen concentration from T250 to P400 and P600 may be associated with the redistribution of fuel-bound nitrogen during sewage sludge pyrolysis and with the relative enrichment of nitrogen retained in the solid as volatile matter is progressively removed. Nitrogen in sewage sludge can be redistributed among char, condensable products, and gaseous species such as NH_3_ and HCN during pyrolysis.

Sulfur was also consistently higher in sewage sludge solids, reaching 1.40 ± 0.24% S at P600, compared with 0.08 ± 0.02% S in green coconut husk biochar. These differences are relevant for product applications and for the interpretation of combustion emissions, since nitrogen- and sulfur-containing compounds in sewage sludge can contribute to NOx and SO_2_ formation during the thermal use of the derived gases or solids.^10^

From an environmental perspective, the retention of N and S in sewage-sludge-derived solids is relevant because subsequent combustion of sewage-sludge char can result in NO and SO_2_ emissions, whose magnitudes depend on the combustion conditions.^33^ During sewage sludge pyrolysis, fuel-bound nitrogen and sulfur are redistributed among the solid, condensable, and gaseous fractions. Nitrogen conversion can generate gaseous NH_3_ and HCN, which are important NOx precursors,^34^ whereas sulfur transformation involves its partitioning among char, tar, and gas, including the formation of gaseous H_2_S.^35^

Sewage sludge can also contain potentially toxic metals that may become concentrated in the resulting biochar as organic matter and volatile components are progressively removed during pyrolysis. However, pyrolysis can simultaneously transform these metals from more mobile fractions into chemically more stable forms, thereby reducing their bioavailability and potential environmental risk.^36^ Therefore, the environmental risk associated with sewage sludge-derived biochar should be evaluated by considering metal speciation and mobility rather than total metal concentration alone.

### 3.2 Energetic Properties and Combustion Behavior of Liquid and Gaseous Products

Table 3 presents the heating values of the raw feedstocks, pellets, torrefied solids, biochars, condensable liquid fractions, and non-condensable gases obtained from torrefaction and pyrolysis. For the solid fraction, green coconut husk consistently showed higher heating values than sewage sludge, reflecting its higher carbon content and lower ash content (Table 1).

**Table 3:** Heating Values of Raw Feedstocks, Pellets, and Products Obtained from Torrefaction and Pyrolysis of Green Coconut Husk and Sewage Sludge^a^

Feedstock	Condition	Solid Fraction (MJ·kg^–1^)	Condensable Liquid Fraction (MJ·kg^–1^)	NCG (MJ·m^–3^)
Green Coconut Husk	Raw feedstock	14.86 ± 1.56^a^	----	----
	Pellet	17.26 ± 1.82^a^	----	----
	T250	18.89 ± 1.99^a^	12.28 ± 0.44^a^	9.38 ± 0.33^a^
	P400	26.06 ± 1.99^b^	15.88 ± 1.02^b^	11.84 ± 0.20^b^
	P600	25.44 ± 2.80^b^	19.12 ± 1.36^c^	12.02 ± 0.30^b^
Sewage Sludge	Raw feedstock	11.88 ± 1.76^a^	----	----
	Pellet	13.84 ± 1.52^a^	----	----
	T250	14.61 ± 1.66^a^	12.18 ± 0.58^a^	8.49 ± 0.34^a^
	P400	17.78 ± 2.29^b^	14.25 ± 1.06^b^	10.25 ± 0.27^b^
	P600	18.34 ± 2.26^b^	16.28 ± 1.58^c^	11.60 ± 0.44^c^

a HHV is reported for the solid and condensable liquid fractions in MJ·kg^–1^, whereas LHV is reported for NCG in MJ·m^–3^. T250 corresponds to torrefaction at 250 °C; P400 and P600 correspond to pyrolysis at 400 and 600 °C, respectively. Values are expressed as mean ± standard deviation. Comparisons were performed among thermal conditions within each feedstock and product fraction. Means followed by the same lowercase letter within the same column and feedstock group do not differ significantly according to Tukey’s test (p < 0.05).

The HHV of green coconut husk increased from 17.26 ± 1.82 MJ·kg^–1^ in the pellets to 18.89 ± 1.99 MJ·kg^–1^ at T250 and reached 26.06 ± 1.99 MJ·kg^–1^ at P400. For sewage sludge, the HHV increased from 13.84 ± 1.52 MJ·kg^–1^ in the pellets to 14.61 ± 1.66 MJ·kg^–1^ at T250 and 18.34 ± 2.26 MJ·kg^–1^ at P600. This difference is consistent with the higher mineral fraction of sewage sludge, which limits the energy density of the solid product even after thermal treatment.^6,17^

The condensable liquid fraction also showed increasing heating values with thermal severity for both feedstocks, consistent with studies reporting that pyrolysis temperature strongly affects the distribution and characteristics of sewage-sludge-derived bio-oil.^37^ For green coconut husk, the HHV increased from 12.28 ± 0.44 MJ·kg^–1^ at T250 to 19.12 ± 1.36 MJ·kg^–1^ at P600, while for sewage sludge, the HHV increased from 12.18 ± 0.58 to 16.28 ± 1.58 MJ·kg^–1^. These results indicate that pyrolysis favored the formation of a more energy-dense condensable liquid fraction than torrefaction. The values obtained for green coconut husk are consistent with bio-oils from lignocellulosic biomass and were close to those reported for green coconut husk bio-oil, with heating values of 15.80 ± 0.16 MJ·kg^–1^ at 400 °C and 16.80 ± 0.18 MJ·kg^–1^ at 600 °C.^5^ A detailed assessment of the chemical composition and specific applications of these liquid products would require dedicated compositional characterization beyond the energetic scope of the present study.

The LHV of the NCG increased with temperature for both feedstocks, indicating the progressive formation of combustible gases during pyrolysis. In green coconut husk, the NCG LHV increased from 9.38 ± 0.33 MJ·m^–3^ at T250 to 12.02 ± 0.30 MJ·m^–3^ at P600. In sewage sludge, the increase was from 8.49 ± 0.34 to 11.60 ± 0.44 MJ·m^–3^. This behavior suggests that higher thermal severity favored gas-phase energy recovery, which is consistent with studies showing that increasing pyrolysis temperature enhances the production of light combustible gases such as H_2_, CO, CH_4_, and hydrocarbons.^5,28^

Figure 2 presents the volumetric composition and estimated species-specific yields of the non-condensable gas species obtained from torrefaction and pyrolysis of green coconut husk and sewage sludge pellets.

**Figure 2:**
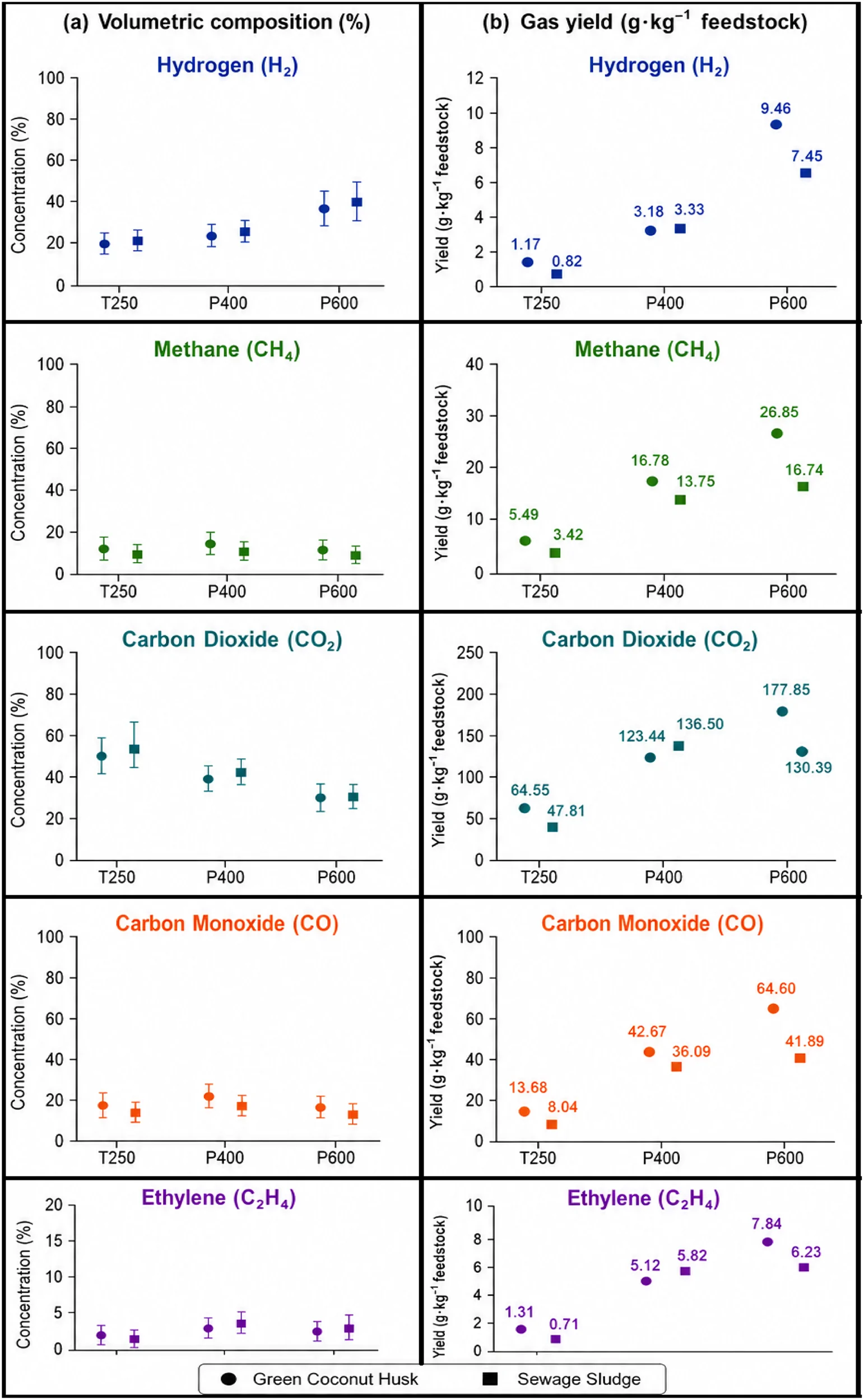
Volumetric composition and estimated species-specific yields of non-condensable gases obtained from torrefaction and pyrolysis of green coconut husk and sewage sludge pellets. (a) Volumetric concentrations of H_2_, CH_4_, CO_2_, CO, and C_2_H_4_ under torrefaction at 250 °C (T250) and pyrolysis at 400 and 600 °C (P400 and P600). (b) Estimated species-specific yields of the corresponding gas species, expressed as g·kg^–1^ of initial feedstock.

As shown in Figure 2a, H_2_ in green coconut husk increased from 19.82 ± 0.98% at T250 to 36.12 ± 2.33% at P600, while CO_2_ decreased from 50.15 ± 3.86% to 31.10 ± 3.55%. A similar trend was observed for sewage sludge, with H_2_ increasing from 20.14 ± 0.82% to 39.22 ± 3.03% and CO_2_ decreasing from 53.8 ± 5.82% to 31.46 ± 2.96%. This behavior indicates a progressive shift in the gas composition toward a more H_2_-rich fraction under higher-temperature pyrolysis conditions. Higher H_2_ formation at higher pyrolysis temperatures has also been reported for pelletized lignocellulosic biomass.^5,28^ The formation of CO and CH_4_ showed a different behavior. CO increased from T250 to P400 for both feedstocks, reaching 21.35 ± 0.77% in green coconut husk and 18.13 ± 0.81% in sewage sludge, but decreased at P600.

CH_4_ reached its highest value at P400 for green coconut husk (14.66 ± 0.44%), while no significant differences were observed among the sewage sludge conditions.

The estimated species-specific yields shown in Figure 2b complement the volumetric composition data by providing an estimate of the amount of each quantified gas species per unit mass of feedstock. For both feedstocks, the estimated yields of H_2_, CO, and CH_4_ were highest at P600, even though the relative concentrations of CO and CH_4_ decreased from P400 to P600. This distinction shows that changes in the volumetric composition do not necessarily correspond to changes in the estimated species-specific yields.

The evolution of the main gaseous species can be related to both the primary decomposition of the feedstock constituents and secondary reactions involving the resulting volatile products. For lignocellulosic biomass, hemicellulose has been reported to generate comparatively higher amounts of CO_2_, cellulose to contribute more strongly to CO formation, and lignin to produce relatively higher proportions of H_2_ and CH_4_.^38,39^ CO_2_ and CO formation are generally associated with decarboxylation and decarbonylation reactions of oxygen-containing structures, respectively, whereas CH_4_ formation is strongly related to the decomposition of lignin-derived structures, including cleavage of methoxyl groups and subsequent formation of methyl radicals.^40^

Recent temperature-resolved analysis of waste lignin further showed that the cleavage of methoxy and ether bonds dominates the main pyrolysis stage, with methoxy demethylation generating methyl radicals that can contribute to CH_4_ formation, while decarbonylation and decarboxylation pathways contribute to CO and CO_2_ release, respectively.^41^ As the thermal severity increases, secondary cracking and dehydrogenation of volatile compounds and carbonaceous intermediates can further contribute to H_2_ formation and alter the relative proportions of CO, CO_2_, and CH_4_. In a recent oil-shale pyrolysis study with steam injection, H_2_ formation was associated with dehydrogenation of aromatic structures, cyclization and aromatization reactions, and aromatic polycondensation, while CH_4_ formation involved the recombination of methyl and hydrogen radicals.^42^

For sewage sludge, the high inorganic fraction may additionally contribute to secondary cracking and reforming of the evolved vapors, since sewage-sludge-derived char and its inherent mineral fraction have been shown to exhibit catalytic activity toward volatile reforming and to promote H_2_ and CO formation.^43^ In addition, gas composition strongly depends on pyrolysis temperature, with H_2_ and CO increasing and CO_2_ decreasing at higher temperatures, while CH_4_ shows a non-monotonic behavior.^44^

These reaction pathways and temperature-dependent behaviors are consistent with the increase in H_2_ and decrease in CO_2_ observed at P600 in the present study as well as with the different behaviors of CO and CH_4_ between P400 and P600. Nevertheless, the measured gas composition represents the net result of several simultaneous primary and secondary reactions, and the individual contributions of each pathway cannot be quantitatively resolved from the present experimental data.

The H_2_/CO ratio, calculated from the gas composition shown in Figure 2a, increased at P600 for both feedstocks, reaching 2.03 for green coconut husk and 2.47 for sewage sludge. For comparison, an H_2_/CO ratio of 1.84 was reported for green coconut husk pellets pyrolyzed at 600 °C under inert conditions, providing a directly comparable pyrolysis-based benchmark.^5^ However, the requirements for downstream syngas utilization are application-dependent rather than defined by a single H_2_/CO ratio. For cobalt-based Fischer–Tropsch synthesis, H_2_/CO ratios close to 2 are commonly employed.^45^ For methanol synthesis, gas suitability is more appropriately evaluated using the stoichiometric number, SN = (H_2_ – CO_2_)/(CO + CO_2_), for which SN = 2 represents the stoichiometric composition.^46^

To place the gaseous products obtained in the present study in the context of recently reported thermochemical systems, Table 4 compares gas composition, hydrogen yield, H_2_/CO ratio, gas heating value, and product yields with selected biomass and sewage sludge pyrolysis and gasification studies.

**Table 4:** Comparison of Gas Composition, Hydrogen Yield, Heating Value, and Product Yields Obtained in the Present Study with Selected Biomass and Sewage Sludge Pyrolysis and Gasification Studies^a^

Feedstock	Process/Main Condition	T (°C)	Gas Composition (vol %)	H_2_ Yield (g H_2_·kg^–1^ feed)	H_2_/CO	Gas Heating Value (MJ·m^–3^)	Product Yields	Reference
Green coconut husk pellets	Pyrolysis/fixed bed	600	H_2_: 36.12 ± 2.33; CO: 17.75 ± 0.48; CO_2_: 31.10 ± 3.55; CH_4_: 12.88 ± 0.37; C_2_H_4_: 2.15 ± 0.15	9.46	2.03	12.02 ± 0.30	Solid: 29.44 ± 1.12%; Liquid: 33.86 ± 1.72%; NCG: 28.66 ± 1.85%; Losses: 8.05 ± 0.52%	Present study
Sewage sludge pellets	Pyrolysis/fixed bed	600	H_2_: 39.22 ± 3.03; CO: 15.88 ± 0.66; CO_2_: 31.46 ± 2.96; CH_4_: 11.08 ± 0.52; C_2_H_4_: 2.36 ± 0.35	7.45	2.47	11.60 ± 0.44	Solid: 37.12 ± 3.32%; Liquid: 33.16 ± 1.90%; NCG: 20.27 ± 2.15%; Losses: 9.45 ± 0.92%	Present study
Green coconut husk pellets	Pyrolysis/fixed bed	600	H_2_: 35.84 ± 4.97; CO: 19.58 ± 0.47; CO_2_: 30.00 ± 4.45; CH_4_: 12.49 ± 0.30	9.56	1.84	10.78 ± 1.22	Biochar: 28.47%; Bio-oil: 28.75%; NCG: 29.11%	^5^
Sewage sludge	Pyrolysis/fixed bed	500	H_2_: 20.80; CO: 8.74; CO_2_: 36.53; CH_4_: 7.36	NR	2.38	NR	Biochar: 58.7%; Bio-oil: 22.4%; Gas: 18.9%	^18^
Sewage sludge	Gasification/fluidized bed	850	H_2_: 33.30 ± 1.19; CO: 26.16 ± 0.74; CO_2_: 20.90 ± 0.42; CH_4_: 11.46 ± 0.75	NR	1.27	12.1	NR	^47^
Sewage sludge	Steam gasification/pilot fluidized bed	800	H_2_: 12.31; CO: 8.38; CO_2_: 20.51; CH_4_: NR	NR	1.47	5.69	Gas: 52.72%; Tar: 11.47%; Char: 35.81%	^48^
Sewage sludge	Combined pyrolysis–gasification–reforming	850	H_2_: 39.0; CO: 13.2; CO_2_: 15.4; CH_4_: 3.5	35.7	2.95	7.27	Syngas: 26.87%; Condensate: 11.41%; Ash: 55.38%; Activated carbon: 6.34%	^49^
Sewage sludge	Pyrolysis/rotary kiln	700	H_2_: ≈38; CO: ≈18; CO_2_: ≈2.5; CH_4_-rich fraction: ≈42	NR	2.10	≈21	Char: 38.3%; Condensable: 14.8%; Gas: 37.0%; adsorbed CO_2_: 9.9%	^50^

a Gas composition is expressed on a volumetric basis (vol %). NR = not reported.

As shown in Table 4, the gaseous products obtained at P600 exhibited H_2_ concentrations and H_2_/CO ratios within the range reported for recent biomass and sewage sludge thermochemical conversion studies. Green coconut husk showed an estimated H_2_ yield of 9.46 g H_2_·kg^–1^ feed, very close to the 9.56 g H_2_·kg^–1^ estimated for green coconut husk pyrolysis,^5^ whereas sewage sludge yielded 7.45 g H_2_·kg^–1^ feed. Higher hydrogen yields, such as 35.7 g H_2_·kg^–1^,^49^ were obtained in combined gasification–reforming systems specifically designed to enhance hydrogen production. The NCG heating values obtained in the present study (11.60–12.02 MJ·m^–3^) were also comparable to those reported for green coconut husk pyrolysis and sewage sludge gasification. Overall, the comparison highlights the competitive gas-phase energy characteristics obtained by direct pyrolysis at 600 °C, while also demonstrating the strong influence of reactor configuration and subsequent reforming or gasification stages on hydrogen production.

Table 5 presents the exhaust gas composition from NCG combustion. CO_2_ concentrations remained relatively stable among conditions, ranging from 3.53 ± 0.35 to 4.21 ± 0.42% vol, indicating comparable overall combustion behavior. In contrast, CO and COREF decreased at P600 for both feedstocks, with CO reaching 25.25 ± 2.53 ppm for green coconut husk and 21.20 ± 2.12 ppm for sewage sludge. This reduction suggests more complete combustion of the NCG produced at the higher pyrolysis temperature, consistent with the higher H_2_ content observed at P600.

**Table 5:** Exhaust Gas Composition from the Combustion of Non-Condensable Gases Obtained from Torrefaction and Pyrolysis of Green Coconut Husk and Sewage Sludge Pellets^a^

Feedstock	Green Coconut Husk			Sewage Sludge		
Condition/Composition	T250	P400	P600	T250	P400	P600
O_2_ (%vol)	18.57 ± 1.86^a^	16.82 ± 1.68^a^	18.41 ± 1.84^a^	19.24 ± 1.92^a^	18.60 ± 1.86^a^	16.81 ± 1.68^a^
CO (ppm)	35.12 ± 3.50^b^	33.97 ± 3.40^b^	25.25 ± 2.53^a^	41.76 ± 4.18^c^	31.68 ± 3.17^b^	21.20 ± 2.12^a^
SO_2_ (ppm)	ND	0.68 ± 0.07^b^	3.90 ± 0.39^c^	ND	0.35 ± 0.03^b^	4.23 ± 0.42^c^
CO_2_ (%vol)	3.73 ± 0.37^a^	3.79 ± 0.38^a^	3.93 ± 0.39^a^	3.60 ± 0.36^a^	3.53 ± 0.35^a^	4.21 ± 0.42^a^
NO_x_ (ppm)	15.69 ± 1.57^a^	17.71 ± 1.77^a^	16.53 ± 1.65^a^	17.44 ± 1.74^a^	16.57 ± 1.66^a^	18.92 ± 1.89^a^
COREF (ppm)	94.18 ± 9.42^b^	85.65 ± 8.56^b^	54.43 ± 5.44^a^	106.32 ± 10.63^c^	85.74 ± 8.57^b^	42.35 ± 4.23^a^
T_gas_ (°C)	213.9 ± 21.4^a^	242.7 ± 24.3^a^	342.1 ± 34.2^b^	231.9 ± 23.2^a^	256.0 ± 25.6^a^	338.9 ± 33.9^b^

a T250 corresponds to torrefaction at 250 °C, whereas P400 and P600 correspond to pyrolysis at 400 and 600 °C, respectively. Values are expressed as mean ± standard deviation. Comparisons were performed among thermal conditions within each feedstock for each parameter. Means followed by the same lowercase letter within the same row and feedstock group do not differ significantly according to Tukey’s test (p < 0.05). ND = not detected under the analytical conditions employed. COREF = CO concentration corrected to the reference oxygen condition; T_gas_ = exhaust gas temperature.

NOx concentrations remained within a narrow range and showed no significant differences among conditions, varying from 15.69 ± 1.57 to 18.92 ± 1.89 ppm. SO_2_, however, increased at P600, reaching 3.90 ± 0.39 ppm for green coconut husk and 4.23 ± 0.42 ppm for sewage sludge. This behavior may be associated with higher thermal severity and with the sulfur content of the feedstocks, especially sewage sludge pellets, which had presented higher sulfur content in Table 1 (0.70 ± 0.12%) than green coconut husk pellets (0.04 ± 0.01%). The higher nitrogen content of sewage sludge pellets, also shown in Table 1 (2.88 ± 0.94%), reinforces the need to monitor nitrogenous emissions during NCG combustion, although NOx did not differ significantly in the present study.

The higher nitrogen and sulfur contents of sewage sludge indicate that nitrogen- and sulfur-containing volatile species may be released during the thermal conversion. However, the analytical configuration used for NCG characterization quantified only H_2_, CO, CO_2_, CH_4_, and C_2_H_4_ and therefore did not allow the individual quantification of nitrogen- and sulfur-containing species in the raw NCG stream. After combustion of the NCG in the flare, the exhaust gases were analyzed for the levels of NO_*x*_ and SO_2_. No significant differences in NO_*x*_ were observed among the evaluated conditions, whereas the level of SO_2_ increased at P600. Therefore, although the present analytical configuration does not allow the speciation or quantitative fate of nitrogen and sulfur in the NCG before combustion to be established, the combustion gas analysis provides information on their potential implications for the energy utilization of the produced gas.

### 3.3 Mass and Energy Balance

Figure 3 shows the product distribution obtained from torrefaction and pyrolysis of green coconut husk and sewage sludge pellets. For both feedstocks, torrefaction at 250 °C favored solid retention, with solid yields of 67.84 ± 6.80% for green coconut husk and 70.26 ± 6.98% for sewage sludge. As the temperature increased to 400 and 600 °C, the solid fraction decreased progressively, reaching 29.44 ± 1.12% for green coconut husk and 37.12 ± 3.32% for sewage sludge at P600.

**Figure 3:**
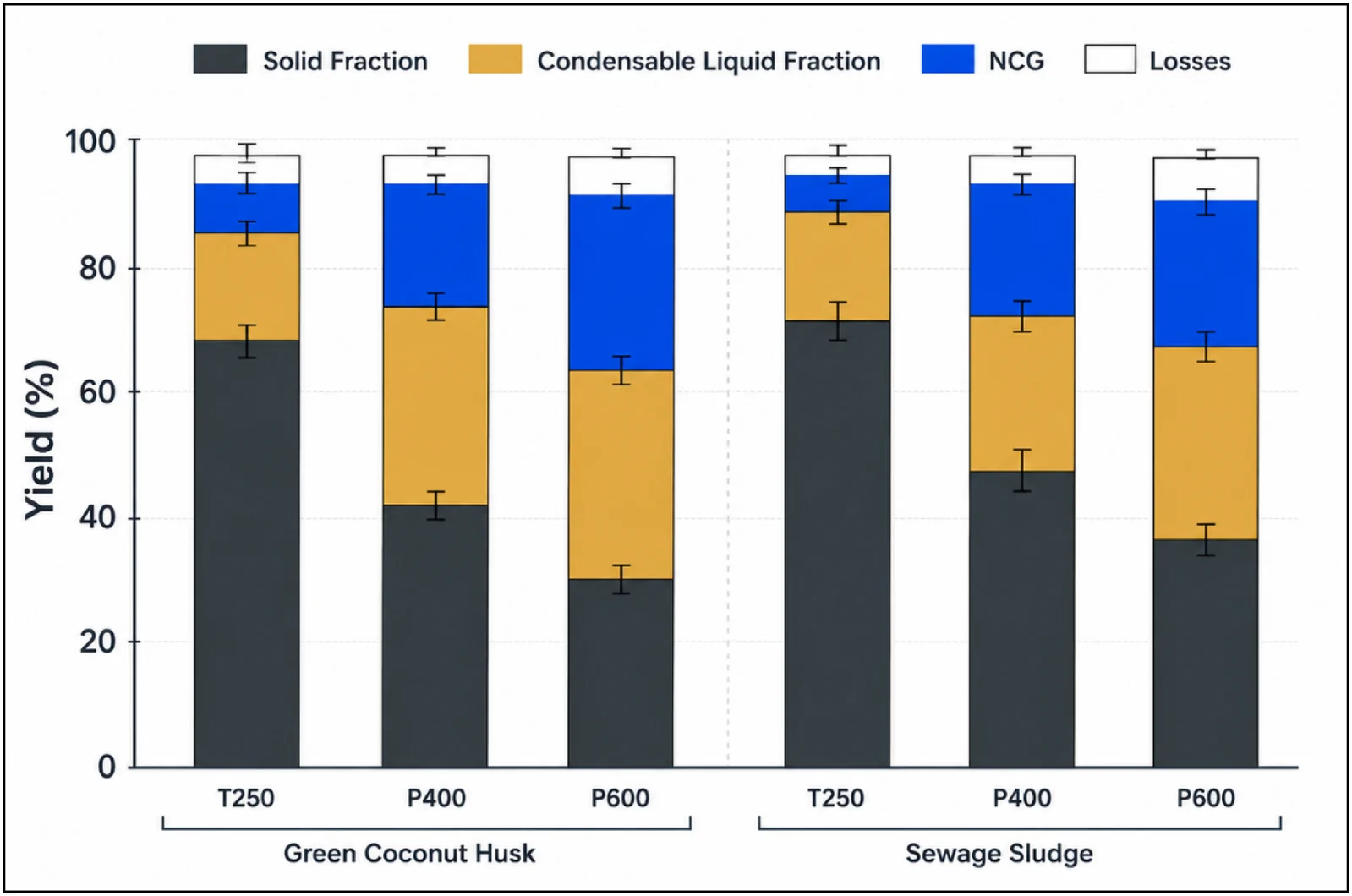
Product distribution from torrefaction and pyrolysis of green coconut husk and sewage sludge pellets. Mass yields of the solid fraction, condensable liquid fraction, non-condensable gases (NCG), and process losses obtained under torrefaction at 250 °C (T250) and pyrolysis at 400 and 600 °C (P400 and P600). The solid fraction corresponds to torrefied solid at T250 and biochar at P400 and P600. Bars represent mean values, and error bars indicate standard deviation.

This reduction in solid yield was accompanied by an increase in the condensable liquid and gaseous fractions, consistent with greater conversion of the feedstock into volatile products under pyrolysis conditions. For green coconut husk, the NCG yield increased from 8.62 ± 0.82% at T250 to 28.66 ± 1.85% at P600, while the NCG yield for sewage sludge increased from 6.08 ± 0.44% to 20.27 ± 2.15%. This behavior is consistent with the expected effect of thermal severity in pyrolysis, in which higher temperatures favor the cracking of organic structures and the release of permanent gases, as reported in previous studies on pelletized lignocellulosic biomass and pyrolysis product yields and energy behavior.^5,28,51,52^

On the basis of the recovered solid, condensable liquid, and NCG fractions, mass-balance closure was 94.78, 92.86, and 91.96% for green coconut husk, and 93.78, 93.15, and 90.55% for sewage sludge at T250, P400, and P600, respectively. The corresponding unaccounted mass losses ranged from 5.22 to 9.45% and generally increased with thermal severity. These losses may include incomplete condensation, retention of condensable compounds in the transfer lines and washing systems, gas leakage, and experimental handling losses. Because the condensation efficiency was not independently determined, its contribution to the unaccounted mass losses could not be quantified separately.

Table 6 presents the energy densification ratio (EDR) and solid energy yield (SEY) of the torrefied solids and biochars. These indices complement the mass balance by quantifying both the solid retention and the increase in energy density resulting from the thermal conversion process.^14^

**Table 6:** Energy Densification Ratio and Solid Energy Yield of Torrefied Solids and Biochars Obtained from Green Coconut Husk and Sewage Sludge Pellets^a^

Feedstock	Green Coconut Husk			Sewage Sludge		
Condition	T250	P400	P600	T250	P400	P600
EDR	1.09	1.51	1.47	1.06	1.28	1.33
SEY (%)	74.25	63.21	43.39	74.17	61.02	49.18

a Energy Densification Ratio (EDR) was calculated as the ratio between the HHV of the solid product and the HHV of the corresponding pellet. Solid Energy Yield (SEY) was calculated as the product of the solid mass yield and EDR. T250 corresponds to torrefaction at 250 °C, whereas P400 and P600 correspond to pyrolysis at 400 and 600 °C, respectively.

For green coconut husk, the highest EDR was observed at P400 (1.51), followed by P600 (1.47), indicating stronger energetic densification under pyrolysis conditions. However, the highest SEY occurred at T250 (74.25%) because torrefaction preserved a larger solid mass fraction. A similar behavior was observed for sewage sludge, with EDR increasing from 1.06 at T250 to 1.33 at P600, while the SEY decreased from 74.17% to 49.18%. These results show that pyrolysis improves the energy density of the remaining solid, whereas torrefaction is more effective at preserving the initial energy in the solid fraction. This trade-off agrees with torrefaction studies reporting that energy yield depends jointly on solid yield and heating value, rather than on HHV alone.^14^

Table 7 presents the energy balance of torrefaction and pyrolysis of green coconut husk and sewage sludge pellets, including the energy recovered in the solid, condensable liquid, and NCG fractions. For both feedstocks, the energy retained in the solid fraction decreased with increasing thermal severity. In green coconut husk, the energy retained in the solid fraction decreased from 12.81 ± 1.86 MJ·batch^–1^ at T250 to 7.49 ± 0.87 MJ·batch^–1^ at P600, while in sewage sludge, it decreased from 10.26 ± 1.55 to 6.81 ± 1.04 MJ·batch^–1^.

**Table 7:** Energy Balance of Torrefaction and Pyrolysis of Green Coconut Husk and Sewage Sludge Pellets^a^

Feedstock	Green Coconut Husk			Sewage Sludge		
Condition	T250	P400	P600	T250	P400	P600
Energy Input (MJ·batch^–1^)						
Feedstock energy	17.26	17.26	17.26	13.84	13.84	13.84
Electrical energy	3.88	6.45	8.13	3.88	6.45	8.13
Total energy input	21.14	23.71	25.39	17.72	20.29	21.97
Energy Output (MJ·batch^–1^)						
Reactor heat losses	3.38	5.81	7.15	3.38	5.81	7.15
Solid fraction	12.81 ± 1.86^b^	10.91 ± 1.20^ab^	7.49 ± 0.87^a^	10.26 ± 1.55^b^	8.44 ± 1.41^ab^	6.81 ± 1.04^a^
Condensable liquid fraction	0.42 ± 0.11^a^	1.87 ± 0.34^b^	3.09 ± 0.49^c^	0.38 ± 0.12^a^	1.11 ± 0.29^a^	2.08 ± 0.54^b^
NCG	0.67 ± 0.07^a^	2.07 ± 0.14^b^	3.82 ± 0.26^c^	0.42 ± 0.03^a^	1.78 ± 0.18^b^	2.69 ± 0.30^c^
Residual system heat losses	3.85	3.05	3.84	3.27	3.15	3.24
Total energy output	21.14	23.71	25.39	17.72	20.29	21.97
Product energy recovery (%)	65.75	62.63	56.72	62.42	55.84	52.71

a Energy values are expressed in MJ·batch^–1^. T250 corresponds to torrefaction at 250 °C, while P400 and P600 correspond to pyrolysis at 400 and 600 °C, respectively. For values reported as mean ± standard deviation, comparisons were performed among thermal conditions within each feedstock. Means followed by the same lowercase letter within the same row and feedstock group do not differ significantly according to Tukey’s test (p < 0.05). NCG = non-condensable gases. Residual system heat losses were calculated by difference to close the global energy balance and were not independently measured. Product energy recovery includes only the energy recovered in the solid, condensable liquid, and NCG fractions; reactor and residual system heat losses are excluded.

The opposite trend was observed for the condensable liquid fraction and NCG. For green coconut husk, the energy recovered in the condensable liquid increased from 0.42 ± 0.11 MJ·batch^–1^ at T250 to 3.09 ± 0.49 MJ·batch^–1^ at P600, while NCG energy increased from 0.67 ± 0.07 to 3.82 ± 0.26 MJ·batch^–1^. Sewage sludge followed the same pattern, with liquid energy increasing from 0.38 ± 0.12 to 2.08 ± 0.54 MJ·batch^–1^ and NCG energy increasing from 0.42 ± 0.03 to 2.69 ± 0.30 MJ·batch^–1^. Similar behavior has been reported in biomass pyrolysis studies, in which higher pyrolysis temperatures reduced the energy retained in biochar and increased the contribution of gaseous products to the overall energy balance.^28,51–53^

To compare the useful energy recovered in the products, the product energy recovery was calculated as the ratio between the sum of the energy recovered in the solid, condensable liquid, and NCG fractions and the total energy input. This indicator decreased with increasing temperature, from 65.75% at T250 to 56.72% at P600 for green coconut husk and from 62.42% to 52.71% for sewage sludge. Therefore, although pyrolysis increased the energy contribution of liquid and gaseous products, torrefaction showed a higher product energy recovery, mainly because of the greater retention of solid mass and energy in the torrefied material.

## 4 Conclusion

This study compared the torrefaction and pyrolysis of green coconut husk and sewage sludge pellets under the same batch-operated experimental system, showing that the feedstock composition strongly controlled product distribution and quality. Green coconut husk behaved as a lignocellulosic residue, with higher contents of volatile matter, fixed carbon, and carbon and lower ash content, favoring the formation of carbon-rich solids and energy-relevant volatile products. Sewage sludge, in contrast, showed an organo-mineral character, with high ash, nitrogen, and sulfur contents, which favored higher solid residue retention but limited the energy quality of the solid products. These characteristics also impose constraints on the downstream utilization of sewage sludge-derived solids. Energy use requires consideration of potential NOx and SO_2_ emissions, whereas soil application would require a specific assessment of nutrient availability, potentially toxic elements, and compliance with applicable regulatory requirements.

Increasing thermal severity from torrefaction at 250 °C to pyrolysis at 400 °C and 600 °C reduced solid yield and increased the formation of condensable liquids and non-condensable gases. In this context, torrefaction was more effective in preserving mass and energy in the solid fraction, as shown by the higher solid energy yield at T250 for both feedstocks. Pyrolysis, especially at 600 °C, promoted greater gas-phase energy recovery and increased H_2_ formation, with H_2_/CO ratios of 2.03 for green coconut husk and 2.47 for sewage sludge. These results demonstrate that increasing pyrolysis temperature substantially altered the gas composition and increased the relative contribution of H_2_ to the gaseous product.

Overall, the main distinction between torrefaction and pyrolysis was the way that energy was retained or redistributed among the products. Torrefaction preserved a larger share of the initial energy in the solid phase, whereas pyrolysis reduced the energy retained in the solid product but increased the contribution of condensable liquids and NCGs. Therefore, under the experimental conditions evaluated, torrefaction favored solid-phase energy retention, while pyrolysis promoted a broader distribution of recovered energy among the solid, liquid, and gaseous products. These findings indicate that the selection of a thermochemical conversion route should consider not only the process severity but also the intrinsic composition of the feedstock and the intended distribution of energy among solid, liquid, and gaseous products. These findings are specific to the 1.0 kg batch-operated system and the experimental conditions evaluated; their extrapolation to larger-scale or continuous systems requires further validation, particularly because heat and mass transfer, vapor residence time, and product recovery may change with the process scale.

## References

[1] Ajien A., Idris J., Sofwan N. M., Husen R., Seli H. (2023). Coconut Shell
and Husk Biochar: A Review of Production and Activation Technology,
Economic, Financial Aspect and Application. Waste Manag. Res..

[2] Agrizzi T., Oliveira M. A., Faria E. V., Santos K. G., Xavier T. P., Lira T. S. (2024). Assessing Coconut Shell Pyrolysis: Biomass Characterization,
Activation Energy Estimation, and Statistical Analysis of Operating
Conditions. Bioresour. Technol. Rep..

[3] Figueiredo A. L., Sousa J. F., Pedroza M. M., Bezerra M. B. D. (2022). Straight Pyrolysis
of Coconut Husk in a Rotating Cylinder Reactor and Product Characterization. Braz. J. Pet. Gas.

[4] Martins T., Sá M. S., Silva M. G., Silva W. R. D., Wisniewski A. (2026). Sustainable Bioproducts of Coconut Husk Biomass—Part
I: Pyrolytic Conversion into Biochar, Bio-Oil, and Pyrolytic Gas. Energy Fuels.

[5] Taveira N. S. S., Serra D. S., Rosa M. D. F., Gondim R. S., Oliveira M. L. M. D., Barros M. D. O., Souza
Filho M. D. S.
M. D., Mattos A. L. A., de Morais S. M., Figuêredo M. C.
B. (2025). Pyrolysis of Green Coconut
Husk Pellets: Process Conditions for the Integrated Production of
Biochar, High-Quality Bio-Oil, and Hydrogen-Rich Gas. Biomass.

[6] Agrafioti E., Bouras G., Kalderis D., Diamadopoulos E. (2013). Biochar Production
by Sewage Sludge Pyrolysis. J. Anal. Appl. Pyrolysis.

[7] Biney M. A., Gusiatin M. Z., Trakal L., Moško J., Pohořelý M. (2025). Utilization of Co-Substrates
in Municipal
Sewage Sludge Co-Pyrolysis: Yields and Characterization of Biochar,
Bio-Oil, and Syngas, with Economic Feasibility Analysis. J. Anal. Appl. Pyrolysis.

[8] Gusiatin M. Z. (2024). Advantages
of Co-Pyrolysis of Sewage Sludge with Agricultural and Forestry Waste. Energies.

[9] Haghighat M., Majidian N., Hallajisani A., Samipourgiri M. (2020). Production
of Bio-Oil from Sewage Sludge: A Review on the Thermal and Catalytic
Conversion by Pyrolysis. Sustain. Energy Technol.
Assess..

[10] Halalsheh M., Shatanawi K., Shawabkeh R., Kassab G., Mohammad H., Adawi M., Ababneh S., Abdullah A., Ghantous N., Balah N., Almomani S. (2024). Impact of Temperature and Residence
Time on Sewage Sludge Pyrolysis for Combined Carbon Sequestration
and Energy Production. Heliyon.

[11] Mohamed B. A., Ruan R., Bilal M., Khan N. A., Awasthi M. K., Amer M. A., Leng L., Hamouda M. A., Vo D.-V. N., Li J. (2023). Co-Pyrolysis of Sewage
Sludge and Biomass for Stabilizing
Heavy Metals and Reducing Biochar Toxicity: A Review. Environ. Chem. Lett..

[12] Pulka J., Manczarski P., Koziel J. A., Białowiec A. (2019). Torrefaction
of Sewage Sludge: Kinetics and Fuel Properties of Biochars. Energies.

[13] Castells B., Paredes R., León D., Amez I. (2025). Kinetic and Thermodynamic
Insights into Sewage Sludge Torrefaction: Energetic Optimization and
Safety Considerations. Energy Nexus.

[14] Manatura K., Samsalee N., Kaewtrakulchai N., Jadsadajerm S., Muangklang E., Jaruwongwittaya T., Huang C.-W. (2025). Optimization of
Torrefaction Parameters for Coconut Shell Using Taguchi Method: Impact
on Torrefaction Performances, Combustion Characteristics, and Thermal
Stability. Therm. Sci. Eng. Prog..

[15] Irawan A., Latifah Upe S., Meity Dwi I. P. (2017). Effect of Torrefaction Process on
the Coconut Shell Energy Content for Solid Fuel. AIP Conf Proc..

[16] Andrade Y. B., de Oliveira Farrapeira R., Schneider J. K., dos Santos Pollidoro A., Freitas L. S., Soares C. M. F., Caramão E. B. (2023). Evaluation
of Torrefaction on the Quality of Bio-Oil Obtained by Pyrolysis of
Green Coconut Wastes. J. Bioeng. Technol. Health.

[17] Trinh T. N., Jensen P. A., Dam-Johansen K., Knudsen N. O., Sørensen H. R. (2013). Influence
of the Pyrolysis Temperature on Sewage Sludge Product Distribution,
Bio-Oil, and Char Properties. Energy Fuels.

[18] Ghodke P.
K., Sharma A. K., Pandey J. K., Chen W.-H., Patel A., Ashokkumar V. (2021). Pyrolysis
of Sewage Sludge for Sustainable Biofuels
and Value-Added Biochar Production. J. Environ.
Manage..

[19] Dissanayaka D. M. N.
S., Udumann S. S., Nuwarapaksha T. D., Atapattu A. J. (2023). Effects of Pyrolysis
Temperature on Chemical Composition of Coconut-Husk Biochar for Agricultural
Applications: A Characterization Study. Technol.
Agron..

[20] Bridgwater A. V. (2012). Review
of Fast Pyrolysis of Biomass and Product Upgrading. Biomass Bioenergy.

[21] Arazo R. O., Genuino D. A. D., de
Luna M. D. G., Capareda S. C. (2017). Bio-Oil Production
from Dry Sewage Sludge by Fast Pyrolysis in an Electrically-Heated
Fluidized Bed Reactor. Sustain. Environ. Res..

[22] Biney M., Gusiatin M. Z. (2024). Biochar from Co-Pyrolyzed Municipal Sewage Sludge (MSS):
Part 1: Evaluating Types of Co-Substrates and Co-Pyrolysis Conditions. Materials.

[23] Ling W., Hong C., Xing Y., Feng W. (2025). Study on the Co-Pyrolysis
Characteristics and Mechanism of Sewage Sludge and Coconut Shell. Thermochim. Acta.

[24] International Organization for Standardization Solid Biofuels—Determination of Moisture Content—Oven Dry Method—Part 1: total Moisture—Reference Method; ISO 18134-1:2022, International Organization for Standardization: Geneva, Switzerland, 2022.

[25] ASTM International Standard Test Methods for Proximate Analysis of Coal and Coke by Macro Thermogravimetric Analysis; ASTM D7582-24, ASTM International: West Conshohocken, PA, 2024.

[26] ASTM International Standard Test Methods for Determination of Carbon, Hydrogen and Nitrogen in Analysis Samples of Coal and Carbon in Analysis Samples of Coal and Coke; ASTM D5373-21, ASTM International: West Conshohocken, PA, 2021.

[27] ASTM International Standard Test Method for Sulfur in the Analysis Sample of Coal and Coke Using High-Temperature Tube Furnace Combustion; ASTM D4239-26, ASTM International: West Conshohocken, PA, 2026.

[28] Ferreira R. M., de Souza L. S., dos Santos G. R., de Sousa F. D., Duarte J. B. F., Carneiro F. O. M., Andrade C. F. D., Serra D. S., de Oliveira M. L. M. (2025). Pyrolysis of Sugarcane Bagasse Pellets:
Influence of
Temperature and Pellet Density on Co-Product Yields. Biofuels Bioprod. Bioref..

[29] Boateng A. A., Daugaard D. E., Goldberg N. M., Hicks K. B. (2007). Bench-Scale Fluidized-Bed
Pyrolysis of Switchgrass for Bio-Oil Production. Ind. Eng. Chem. Res..

[30] Pulka J., Manczarski P., Stępień P., Styczyńska M., Koziel J. A., Białowiec A. (2020). Waste-to-Carbon: Is the Torrefied
Sewage Sludge with High Ash Content a Better Fuel or Fertilizer?. Materials.

[31] Tang S., Zheng C., Zhang Z. (2018). Effect of Inherent Minerals on Sewage
Sludge Pyrolysis: Product Characteristics, Kinetics and Thermodynamics. Waste Manage..

[32] Feng H., Zhou S., Su H., Li R., Wang S. (2026). Insight into
the Influence Mechanism of Inherent Ash in Pyrolysis Recycling of
Sewage Sludge: Dual Roles of Catalysis and Physical Barrier. J. Anal. Appl. Pyrolysis.

[33] Xu K., Deng S., Zheng K., Liu B., Xia Z., Wang X., Tan H., Zhang X., Du Z. (2026). Investigation
on the Emission Characteristics of NO and SO_2_ in the Sewage
Sludge Char Co-Combustion with Biomass. J. Environ.
Manage..

[34] Wei L., Wen L., Yang T., Zhang N. (2015). Nitrogen Transformation during Sewage
Sludge Pyrolysis. Energy Fuels.

[35] Zhang J., Zuo W., Tian Y., Chen L., Yin L., Zhang J. (2017). Sulfur Transformation
during Microwave and Conventional Pyrolysis of Sewage Sludge. Environ. Sci. Technol..

[36] Cui Z., Xu G., Ormeci B., Liu H., Zhang Z. (2022). Transformation and
Stabilization of Heavy Metals during Pyrolysis of Organic and Inorganic-Dominated
Sewage Sludges and Their Mechanisms. Waste Manage..

[37] Huang F., Yu Y., Huang H. (2018). Temperature
Influence and Distribution of Bio-Oil from
Pyrolysis of Granular Sewage Sludge. J. Anal.
Appl. Pyrolysis.

[38] Chen D., Cen K., Zhuang X., Gan Z., Zhou J., Zhang Y., Zhang H. (2022). Insight into Biomass
Pyrolysis Mechanism Based on Cellulose, Hemicellulose,
and Lignin: Evolution of Volatiles and Kinetics, Elucidation of Reaction
Pathways, and Characterization of Gas, Biochar and Bio-Oil. Combust. Flame.

[39] Zhang S., Mei Y., Lin G. (2024). Pyrolysis Interaction of Cellulose, Hemicellulose and
Lignin Studied by TG-DSC-MS. J. Energy Inst..

[40] Wang L., Fang Y., Yin J., Li X., Jiang J., Zhang Y., Yang H. (2022). Fast Pyrolysis of Guaiacyl-Syringyl
(GS) Type Milled Wood Lignin: Product Characteristics and CH_4_ Formation Mechanism Study. Sci. Total Environ..

[41] Yue Q., Guo Y., Chen S., Chen B. (2026). Investigation of Functional Group
Changes and Reaction Mechanisms during the Thermochemical Conversion
of Waste Lignin. AIP Adv..

[42] Kang Z., Huang D., Zhao J., Fan S., Yang D., Lu Y. (2025). Hydrogen Transfer and Reaction Mechanism during In Situ Pyrolysis
of Fushun Oil Shale with Steam Injection. Fuel.

[43] Bai J., Lv Q., Fu X., Chen J., Wang H., Lin S., Zhou X. (2022). Catalytic
Reforming of Sewage Sludge Pyrolysis Products over the
Self-Derived Char. J. Anal. Appl. Pyrolysis.

[44] Moško J., Pohořelý M., Skoblia S., Beňo Z., Jeremiáš M. (2020). Detailed Analysis
of Sewage Sludge
Pyrolysis Gas: Effect of Pyrolysis Temperature. Energies.

[45] Gavrilović L. (2021). Fischer–Tropsch Synthesis over an Alumina-Supported Cobalt
Catalyst in a Fixed-Bed Reactor: Effect of Process Parameters. Catal. Today.

[46] Nestler F. (2020). Kinetic Modelling of Methanol Synthesis over Commercial
Catalysts:
A Critical Assessment. Chem. Eng. J..

[47] Migliaccio R., Brachi P., Montagnaro F., Papa S., Tavano A., Montesarchio P., Ruoppolo G., Urciuolo M. (2021). Sewage Sludge Gasification
in a Fluidized Bed: Experimental Investigation and Modeling. Ind. Eng. Chem. Res..

[48] Chen Y.-H., Ngo T. N. L. T., Chiang K.-Y. (2021). Enhanced Hydrogen
Production in Co-Gasification
of Sewage Sludge and Industrial Wastewater Sludge by a Pilot-Scale
Fluidized Bed Gasifier. Int. J. Hydrogen Energy.

[49] Gebhard W., Zant S., Neidel J., Apfelbacher A., Daschner R. (2025). Conversion of Sewage Sludge with
Combined Pyrolysis
and Gasification via the Enhanced Carbon-To-X-Output Technology. Biomass.

[50] Freda C., Fanelli E., Romanelli A., Valerio V., Le Pera A., Sellaro M., Cornacchia G., Braccio G. (2026). Methane-Rich Syngas
from Pyrolysis of Sewage Sludge with Sorbent/Catalyst. Biomass.

[51] Awad M. I., Makkawi Y., Hassan N. M. (2024). Yield and
Energy Modeling for Biochar
and Bio-Oil Using Pyrolysis Temperature and Biomass Constituents. ACS Omega.

[52] Ben H., Ragauskas A. J. (2011). Pyrolysis of Kraft Lignin with Additives. Energy Fuels.

[53] Yadav G., Lamers P. (2026). Process Feasibility
Analysis of Waste Biomass Valorization
to Biochar and Bio-Oil via Slow and Fast Pyrolysis. Energy Fuels.

